# A Novel Case of Acute Respiratory Distress Syndrome Following Radiofrequency Ablation for Atrial Fibrillation

**DOI:** 10.7759/cureus.39052

**Published:** 2023-05-15

**Authors:** Gilliann D Minviel, Heather Gosnell, Mathew Karivelil, Israel C Ugalde, Sherri Huang

**Affiliations:** 1 Internal Medicine, USF Health, Tampa, USA; 2 Pulmonary and Critical Care Medicine, USF Health, Tampa, USA; 3 Internal Medicine-Pediatrics, USF Health, Tampa, USA

**Keywords:** pneumopericardium, pneumomediastinum, rfa, catheter ablation, atrial fibrillation, ards, radiofrequency ablation

## Abstract

Radiofrequency ablation (RFA) is a minimally invasive cardiac catheterization procedure employed in patients whose atrial fibrillation (AF) is not well-controlled on medical therapy. While serious complications after the RFA are uncommon, we present the unique case of a 71-year-old male who suffered from acute respiratory distress syndrome (ARDS) and pneumomediastinum post-procedure. He presented to the ED with dyspnea, non-massive hemoptysis, and fever three days following RFA. Admission CT thorax demonstrated patchy ground glass opacities (GGOs) and stable fibrotic changes. He was admitted for suspected pneumonia, however, he failed to significantly improve on broad-spectrum antibiotics. Bronchoscopy found blood in proximal airways, however, lavage with serial aliquots were without worsening hemorrhage, ruling out suspected diffuse alveolar hemorrhage. Cytology resulted in rare iron polymorphonuclear neutrophils and no malignant cells. With worsening clinical status, the patient was eventually intubated. Repeat CT thorax showed new moderate pneumopericardium, small pneumomediastinum, and progressed GGOs. The respiratory course continued to worsen, and the patient passed away approximately one month after admission. We also present a brief literature review with the aim of identifying prognostic risk factors regarding post-RFA ARDS development. Additionally, this case identifies a novel complication of RFA, as post-procedural pneumomediastinum has not been previously described.

## Introduction

Conventional management of atrial fibrillation (AF) aims to alleviate symptoms with rate- and rhythm-controlling medications while reducing the risk of tachycardia-mediated cardiomyopathy and stroke. In patients who are not adequately controlled on medical therapy, catheter ablation is a treatment option that can provide prolonged symptomatic relief in up to 60-75% of cases [1]. Determining optimal candidates for catheter ablation depends on the nature of AF because the efficacy is lower in patients with persistent AF compared to paroxysmal AF [2]. Thus, in patients with persistent AF, catheter ablation is typically reserved for cases that fail or who cannot tolerate drug therapy [3]. 

Several types of catheter ablation techniques exist, including radiofrequency ablation (RFA) and cryothermal ablation. Across several studies including the FreezeAF trial, both approaches have been found to have comparable efficacy and safety profiles [1,4,5]; the targeted ectopic foci are most often located near the pulmonary veins [6]. 

Serious complications of RFA are rare, occurring only in 4% of cases, more than half of which are complications related to vascular access [7]. Other reported complications include pulmonary vein stenosis, cardiac tamponade, pericarditis, transient ischemic attack, stroke, phrenic nerve paralysis, hemothorax, esophageal perforation, and atrio-esophageal fistula [7,8]. To our knowledge, very few cases of acute respiratory distress syndrome (ARDS) following RFA have been reported. 

Of the reported cases, one immediately followed the procedure and the other occurred one year post-RFA and was related to bilateral pulmonary vein stenosis [9,10]. Here we describe the unique clinical course of a patient who developed ARDS several days following RFA under general anesthesia and pulmonary vein stenosis was notably absent. This case was previously presented as a poster at the CHEST Congress 2022 conference. 

## Case presentation

**Figure 1:**
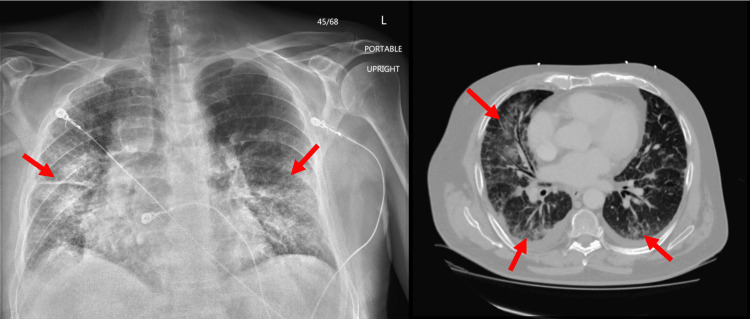
Left: Chest X-ray with nonspecific diffuse airspace opacity throughout the lungs bilaterally with sparing towards the left lung apex compared to chest X-ray 5 days prior. Right: CT chest with interval development of patchy ground-glass opacities bilaterally with baseline extensive fibrotic changes compared to CT chest 18 months prior.

This patient was a 71-year-old male with a history of persistent AF on chronic dabigatran, obstructive sleep apnea, mitral valve prolapse, and 84-pack-year history of prior smoking. Three days after an uncomplicated trans-septal RFA procedure, he presented with dyspnea, non-massive hemoptysis, and subjective fever. In the emergency department, he required increasing amounts of supplemental oxygen up to 4 L via nasal cannula but was otherwise hemodynamically stable. Admission chest X-ray and CT thorax showed interval development of ground glass opacities (GGOs) and stable fibrotic changes (Figure 1) compared to previous chest imaging.

He was admitted to the cardiac intensive care unit (ICU) due to worsening hypoxia and concerns for post-RFA pneumonia. He was initially treated with amoxicillin-clavulanic acid and azithromycin for suspected pneumonia. The antibiotic regimen was later expanded to cefepime and azithromycin on hospital day 2 per infectious disease recommendations. Due to ongoing hemoptysis during this time, dabigatran was held. 

On hospital day 3, respiratory status declined as evidenced by worsening hypoxemia and hypercapnia with persistent non-massive hemoptysis. Non-invasive ventilation was utilized, and pulmonology was consulted due to concerns for diffuse alveolar hemorrhage (DAH) and evaluation for diagnostic bronchoscopy. He was started on methylprednisolone 250 mg IV followed by prednisone 40 mg daily and with clinical improvement on hospital day 4. Supplemental oxygen was decreased to 3 L nasal cannula, and bronchoscopy was temporarily deferred. 

Suddenly, he re-developed non-massive hemoptysis on hospital day 5 and required escalation to a heated high-flow nasal cannula for persistent hypoxia. A portable chest X-ray showed unchanged bilateral infiltrates. After a discussion with the patient and his wife, he was electively intubated and a bronchoscopy was performed out of growing concern for DAH, but the findings were nondiagnostic. Although blood was visualized in the proximal airways, bronchoalveolar lavage with serial aliquots did not show progressive sanguineous samples. Cytology showed no malignant cells and rare macrophages with minimal stainable iron in the cytoplasm making DAH unlikely. He remained intubated post-procedurally and underwent aggressive diuresis. On day 7 of admission, his respiratory status improved and he was extubated to 6 L via a high-flow nasal cannula. Given the improvement in clinical status and cessation of hemoptysis, therapeutic anticoagulation was restarted with heparin infusion and subsequently transitioned to dabigatran. 

**Figure 2:**
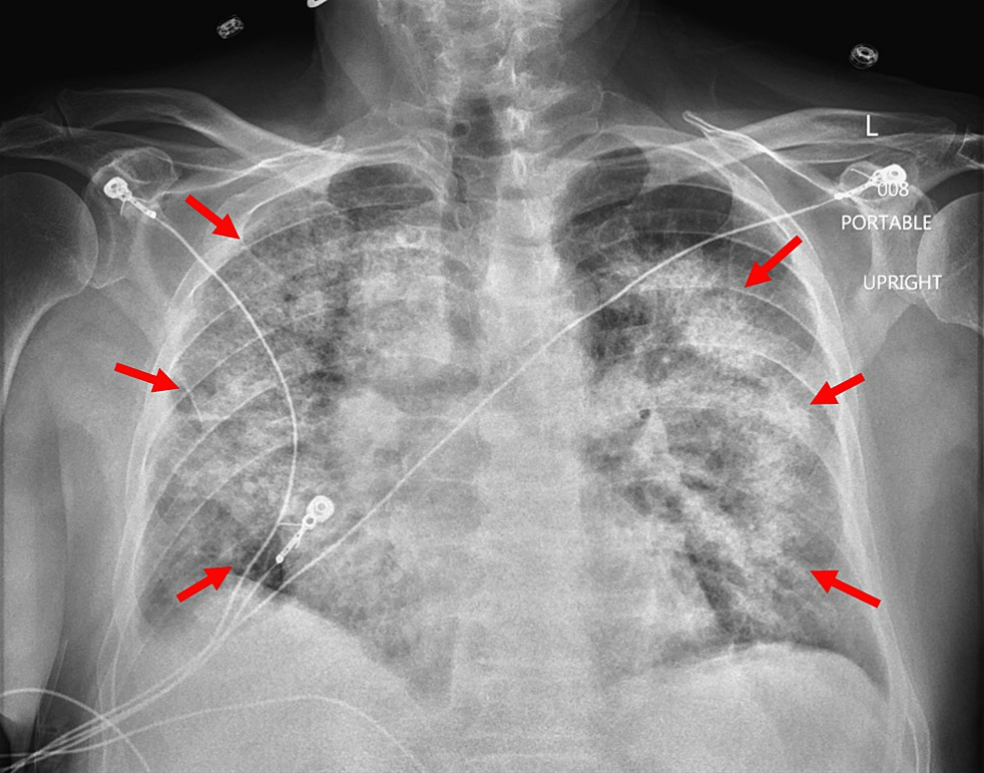
Chest X-ray demonstrating the progression of bilateral infiltrates.

Unfortunately, he experienced worsening dyspnea and hypoxia on hospital day 10, and was placed on non-invasive positive pressure ventilation with alternating heated high flow nasal cannula and bilevel positive airway pressure. At this time, a respiratory viral panel was conducted with negative findings. A stat portable chest X-ray demonstrated extensive, worsened bilateral infiltrates (Figure 2).

The patient developed worsening bilateral infiltrates and hypoxia, and his arterial blood gas revealed a partial pressure of oxygen (PaO2)/fraction of inspired oxygen (FiO2) ratio of 72, all consistent with severe ARDS. Pulmonology recommended expanding and extending the course of antibiotics with piperacillin-tazobactam, along with continued steroids and diuresis. After the patient remained unimproved by hospital day 11, corticosteroids were increased to 60 mg daily. He was transferred from the coronary care unit to the medical ICU for further care. Infectious disease broadened antimicrobial coverage to include meropenem and micafungin. The patient’s hypoxia continued to worsen despite these measures and maximized oxygen therapy on 60 L on a high-flow nasal cannula. Ultimately, he required invasive mechanical ventilation on hospital day 15. 

**Figure 3:**
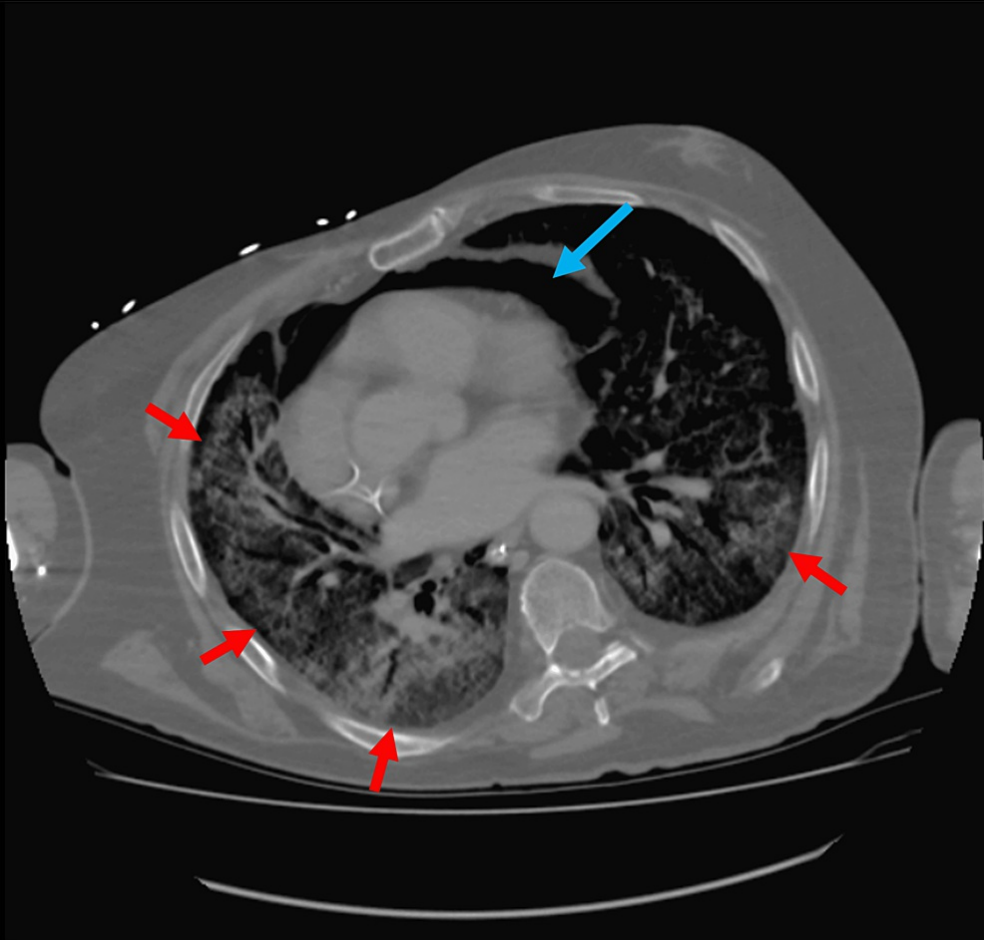
CT chest with a marked progression of bilateral opacities (red arrows) and development of pneumopericardium (blue arrow).

By hospital day 18, he had finished a 7-day course of meropenem with no growth in the blood cultures and stagnant respiratory status. On day 20, CT Chest with contrast revealed new moderate pneumopericardium and new small pneumomediastinum along with progressed, extensive GGOs bilaterally (Figure 3).

Cardiothoracic surgery advised these new findings were likely related to barotrauma from positive pressure ventilation in the setting of severe ARDS. Surgical intervention was not indicated. Despite medical management and continued careful adjustment of ventilator settings, the patient developed a right-sided pneumothorax on day 23, and a percutaneous chest thoracostomy tube was placed. After ongoing goals of care discussions with family, he was transitioned to comfort measures on day 24 and transferred to hospice care where he subsequently succumbed to his illnesses on day 29. 

## Discussion

To our knowledge, only two cases of post-RFA ARDS have been reported prior to this patient, who additionally developed pneumopericardium and pneumomediastinum during his hospital course. In the Novak and Segal case, a 65-year-old previously healthy male developed dyspnea and bilateral lung basilar crackles several hours after a pulmonary vein isolation for paroxysmal AF [9]. Chest X-ray revealed bilateral pulmonary infiltrates, and infectious and vasculitis workups were negative. He was treated with intravenous furosemide, antibiotics, non-steroidal anti-inflammatory drugs, and non-invasive ventilation. He eventually recovered and was discharged home after a week. In the Spadaro et al. case, a 40-year-old heavy tobacco user underwent pulmonary vein isolation for atrial fibrillation [10]. One year later, the patient presented to the ICU with massive hemoptysis and developed severe hypoxia with patchy bilateral infiltrates on chest X-ray. Chest CT angiography of the pulmonary arteries on hospital day 7 showed severe stenosis of the right superior pulmonary vein and 50% stenosis of both left pulmonary veins. The patient underwent stent replacement of the stenotic veins and was extubated. The worsening clinical course of our patient requiring intubation is most consistent with the Spadaro et al. case, where both patients had a significant history of heavy smoking.

Management of ARDS includes supportive ventilative care and evaluation for the underlying cause. Care for this patient included both diagnostic evaluation and standard management of ARDS by optimization of peak end-expiratory pressure (PEEP), airway clearance, and attempts at early physical therapy [11]. In the medical ICU, he was intubated for refractory hypoxia despite non-invasive positive pressure ventilation efforts with continued optimization of his PEEP and lower tidal volumes delivered. However, despite these measures, he never made a significant clinical improvement. His comorbidities of prolonged and significant cigarette smoking, obstructive sleep apnea with positive airway pressure intolerance, and mitral valve disease may have contributed to a poor prognosis. Differential diagnoses for his hemoptysis, dyspnea, and fevers included pneumonia, aspiration pneumonitis, pulmonary vein stenosis, DAH, and malignancy. Pneumonia was less likely given minimal improvement on broad-spectrum antibiotics and no growth from bronchoalveolar lavage for bacterial, fungal, and acid-fast bacillus culture. He was initiated on steroids for presumed pneumonitis. The patient initially improved, however, he re-developed moderate hemoptysis and respiratory decompensation. Aspiration pneumonitis was a possibility, as the risk of pulmonary aspiration has been shown to be highest perioperatively, especially during induction of anesthesia [12]. There was no documented history of the patient coughing or choking with meals following RFA, and the temporal development of symptoms makes this diagnosis less likely. However, the ability to confidently rule out aspiration pneumonitis is limited by history. During the bronchoscopy, sequential aliquot samples were without increasing sanguinity, and lavage was without hemosiderin-laden polymorphonuclear cells, making DAH less likely. There was no secondary evidence of pulmonary vein stenosis on CT thorax with contrast, and the timing of symptom presentation is inconsistent with the Spadaro et al. case [10]. Compared to previous case reports, the temporal development of his symptoms is consistent with the Novak and Segal case [9]. 

Another complication of his hospital course was the development of pneumomediastinum and pneumopericardium initially in the absence of pneumothorax. Pneumomediastinum may be categorized as either traumatic, after a known insult, or spontaneous when no traumatic or iatrogenic injury occurred. Additionally, spontaneous pneumomediastinum may be divided between primary, in the absence of any known lung disease, or secondary when there is lung disease present. The pathophysiology of spontaneous pneumomediastinum is thought to be due to air leaking from the rupture of alveoli that travel along the bronchovascular sheath to the mediastinum or sub-pleural space. This was first described by Charles Macklin in 1939 and termed the Macklin effect [13]. Air in the confined space can accumulate and cause significant compression of mediastinal structures such as the heart. 

If the etiology of the pneumomediastinum is thought to be due to a known pneumothorax, a chest thoracostomy tube should be inserted into the affected pleural space. If no pneumothorax is identified, a mediastinal thoracostomy tube should be considered in the presence of tension pneumomediastinum, manifested as refractory hypotension with right ventricular collapse, to help relieve the life-threatening pressure [14]. Pneumomediastinum has previously been identified as a poor prognostic factor for adults with ARDS secondary to COVID-19, however, it is a less frequent complication in non-COVID-19 ARDS [15]. In our patient’s case, pneumomediastinum and pneumopericardium likely occurred secondary to barotrauma from prolonged positive-pressure ventilation. Cardiothoracic surgery did not recommend surgical intervention during their initial evaluation, however, a chest thoracostomy tube was placed after the development of a pneumothorax. 

Previous studies have associated older age, female gender, history of congestive heart failure, and duration of RFA with increased risk of post-RFA complications [6,14]. However, as these studies do not include post-RFA ARDS as a complication due to its presumably low prevalence, it is unclear if these risk factors bear relevance to our patient’s case. While it appears post-RFA ARDS may be rare, ongoing analysis of patient epidemiology, pre-existing medical conditions, and areas of embolization may provide both risk and prognostic stratification for patients with regard to ARDS development [16].

## Conclusions

ARDS is a rare but important complication of RFA seldom documented in the literature. The clinical presentation and extensive workup ruled out known complications of RFA. Despite the multidisciplinary approach to treatment, this patient failed to make significant improvement and eventually succumbed to his disease after one month. Due to the infrequent case reports of post-RFA ARDS, further research on pathophysiology and risk factors is needed to accurately determine a patient’s risk. Ongoing reports of patient epidemiology, pre-existing medical conditions, and RFA procedure details may provide risk and prognostic stratification for ARDS development.
